# Imaging Mitochondrial Flux in Single Cells with a FRET Sensor for Pyruvate

**DOI:** 10.1371/journal.pone.0085780

**Published:** 2014-01-21

**Authors:** Alejandro San Martín, Sebastián Ceballo, Felipe Baeza-Lehnert, Rodrigo Lerchundi, Rocío Valdebenito, Yasna Contreras-Baeza, Karin Alegría, L. Felipe Barros

**Affiliations:** 1 Centro de Estudios Científicos (CECs), Valdivia, Chile; 2 Universidad Austral de Chile, Valdivia, Chile; University of Oldenburg, Germany

## Abstract

Mitochondrial flux is currently accessible at low resolution. Here we introduce a genetically-encoded FRET sensor for pyruvate, and methods for quantitative measurement of pyruvate transport, pyruvate production and mitochondrial pyruvate consumption in intact individual cells at high temporal resolution. In HEK293 cells, neurons and astrocytes, mitochondrial pyruvate uptake was saturated at physiological levels, showing that the metabolic rate is determined by intrinsic properties of the organelle and not by substrate availability. The potential of the sensor was further demonstrated in neurons, where mitochondrial flux was found to rise by 300% within seconds of a calcium transient triggered by a short theta burst, while glucose levels remained unaltered. In contrast, astrocytic mitochondria were insensitive to a similar calcium transient elicited by extracellular ATP. We expect the improved resolution provided by the pyruvate sensor will be of practical interest for basic and applied researchers interested in mitochondrial function.

## Introduction

Mitochondria are the main generators of cellular ATP and are also important sites for metabolite degradation and synthesis. Mitochondria participate in the production and detoxification of reactive oxygen species, in the regulation of calcium signaling, apoptosis, necrosis and autophagy, playing roles in degenerative diseases and cancer progression. Mitochondrial function can be approached at high resolution using fluorescence microscopy through the estimation of mitochondrial calcium, potential, pH, NAD(P)H autofluorescence, etc. However, these parameters are only indirectly related to mitochondrial metabolism, and may be ambiguous: for example, higher mitochondrial calcium concentration and mitochondrial depolarization may be observed when cells are active but also when cells are dying [1].

The speed of mitochondrial metabolism, i.e. the metabolic rate, is typically estimated as the rate of oxygen consumption, measured in cell populations or purified mitochondria with the aid of extracellular oxygen electrodes or sensors. Alternatively, mitochondrial metabolism can be assessed with isotopically-labeled metabolites, which are detected as CO_2_ released into the medium, or through the steady-state enrichment of the isotopic label in intermediate metabolites, which can be detected non-invasively by NMR spectroscopy. A common limitation of these techniques is a requirement for cell populations, which makes it difficult to ascertain the contribution of individual cell types within complex tissues, tumors or cell cultures. In addition, some existing methods have low temporal resolution, which precludes the study of rapid metabolic phenomena, such as are thought to occur in the brain. Much of what is known about the function and dysfunction of mitochondria has stemmed from the study of purified organelles [1], but mitochondria do not work in isolation, and their function is known to be modulated by cytosolic molecules and interactions with other organelles, which are lost during the purification procedure.

In this article we introduce a method for the estimation of mitochondrial flux in single intact cells, in real-time, using fluorescence microscopy. The method is based on a novel genetically-encoded FRET nanosensor for pyruvate, which in addition, allows the estimation of intracellular pyruvate levels, cellular pyruvate transport and the rate of glycolytic pyruvate production.

## Results

### PdhR flanked by the FRET pair mTFP-Venus reports pyruvate concentration

**Figure 1:**
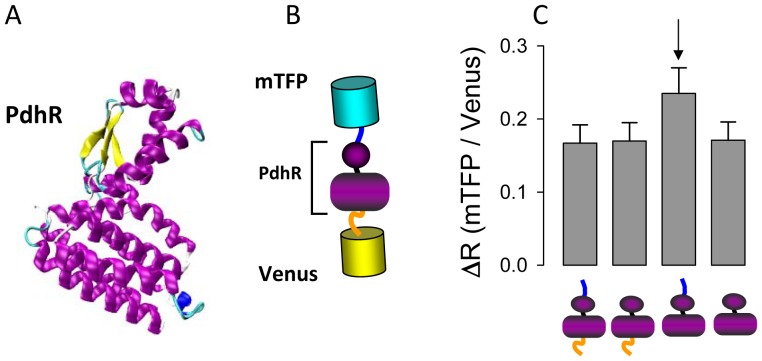
Pyronic, a FRET pyruvate sensor based on the transcriptional regulator PdhR. A. 3D-structure of PdhR from *Escherichia coli* predicted by analogy to LldR and FadR, (http://www.fiserlab.org/servers_table.htm). B. Sensor design: the transcriptional regulator PdhR was sandwiched between the fluorescent proteins mTFP (FRET donor) and Venus (FRET acceptor), with synthetic peptides separating the proteins (blue and orange linkers). C. Effect of 5 mM pyruvate on the fluorescence ratio of four variants of the pyruvate sensor. The most responsive of the constructs (arrow), was termed Pyronic and used for further characterization and cell measurements.

**Figure 2:**
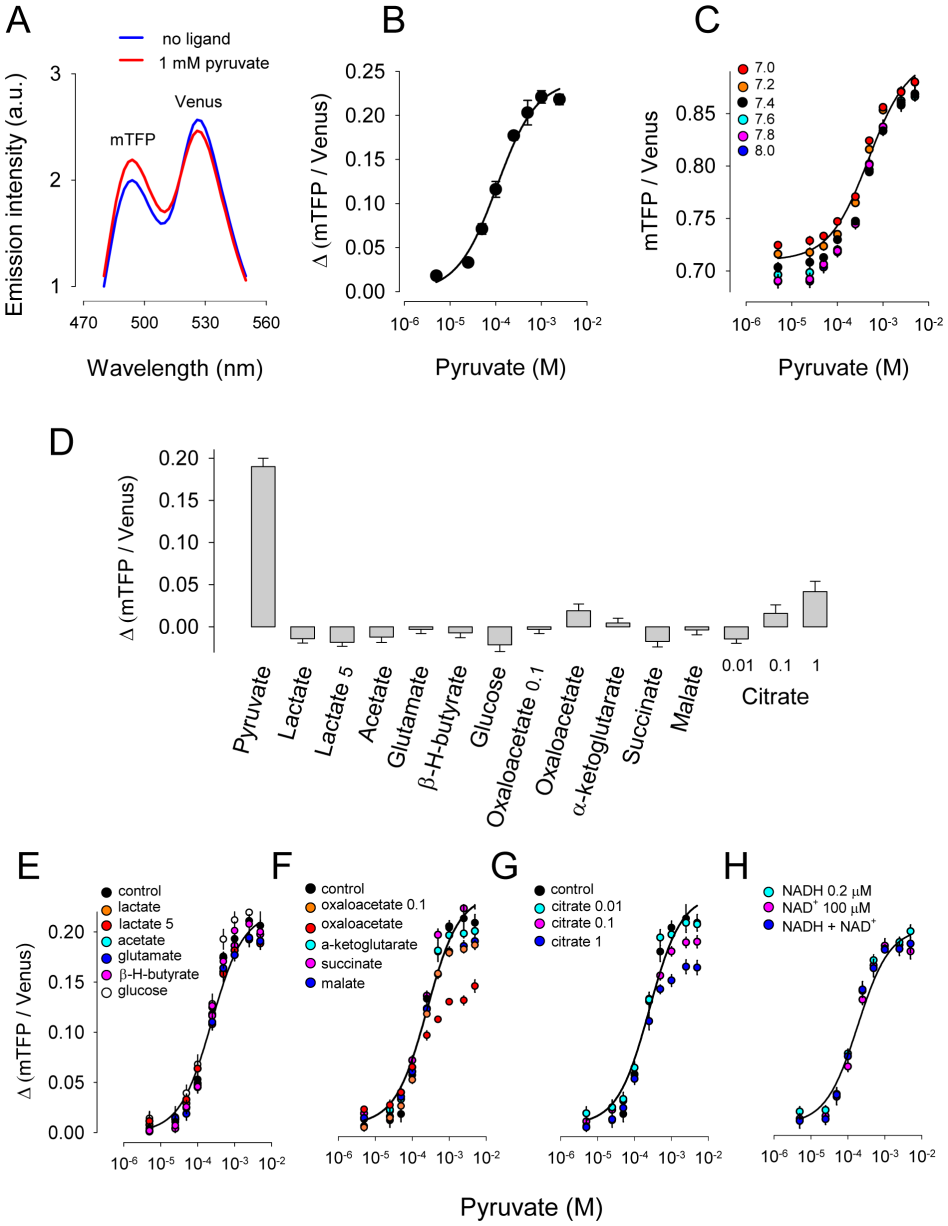
*In vitro* characterization of Pyronic. A. Emission spectra in the absence and presence of 1(at 462 nm excitation) was measured at increasing concentrations of pyruvate. The continuous line corresponds to the best fit of a rectangular hyperbola to the data, with an apparent dissociation constant (K_D_) value of 107±13 µM and a maximum change in fluorescence ratio (ΔRmax) of 24±0.7%. C. Pyruvate dose-response curves were measured at the indicated pH values. The continuous line shows the best fit of a rectangular hyperbola to the data at pH 7.2. D. Response of Pyronic to a panel of metabolites at 1 mM, unless otherwise specified. E-F-G. Pyruvate dose-response curves were measured in the absence and presence of a panel of metabolites at 1 mM, unless otherwise specified. The best fits of rectangular hyperbolae to control data are shown. H. Pyruvate dose-response curves were measured in a reduced environment (0.2 µM NADH), an oxidized environment (100 µM NAD^+^) or an intermediate environment (0.2 µM NADH plus 100 µM NAD^+^). The best fit of a rectangular hyperbola to the latter is shown.

PdhR is a bacterial transcriptional regulator that controls the expression of the pyruvate dehydrogenase gene in response to pyruvate [2]. In order to make a FRET nanosensor for pyruvate, PdhR from *Escherichia coli* (Fig. 1A) was selected as scaffold protein and flanked with the fluorescent proteins mTFP and Venus, as respective FRET donor and acceptor (Fig. 1B). Four variants of the hybrid protein were built, with and without linkers. The corresponding DNA and amino acid sequences are described in Figure S1. The ratio between the fluorescence intensity of mTFP and Venus (R) was measured in the absence and presence of 5 mM pyruvate. The pyruvate-dependent change in the ratio (ΔR) of the four variants is shown in Fig. 1C. The variant with the largest response was termed Pyronic (Pyruvate Optical Nano Indicator from CECs) and was characterized in more detail. As shown in Fig. 2A, exposure of Pyronic to pyruvate caused an increase in mTFP fluorescence intensity and a decrease in Venus fluorescence intensity, consistent with a decrease in FRET efficiency. Exposure to increasing concentrations of pyruvate showed that Pyronic responds to pyruvate between 10 µM to 1 mM, with a K_D_ of 107±13 µM and a maximum change in fluorescence ratio of approximately 20% (Fig. 2B). This K_D_ in the micromolar range is consistent with gel retardation assays with purified PdhR [2]. Pyruvate sensing was not significantly affected by pH in the physiological or pathological range (Fig. 2C). The sensor was not affected by millimolar levels of lactate, acetate, glutamate, beta-hydroxybutyrate, glucose, alpha-oxoglutarate, succinate or malate, either in the absence (Fig. 2D) or presence of pyruvate (Fig. 2E–F). Pyruvate detection was insensitive to the redox ratio (Fig. 2H). A small effect was observed for citrate at 1 mM but not at 100 µM, the concentration observed in mammalian cytosol [3]–[5](Figs. 2D and 2G). Oxaloacetate at 1 mM produced a small change in fluorescence ratio, but not at 100 µM, a concentration well above the low micromolar level present in mammalian cells [3](Figs. 2D and 2F).

### Pyronic reports cytosolic concentrations of pyruvate in mammalian cells

**Figure 3:**
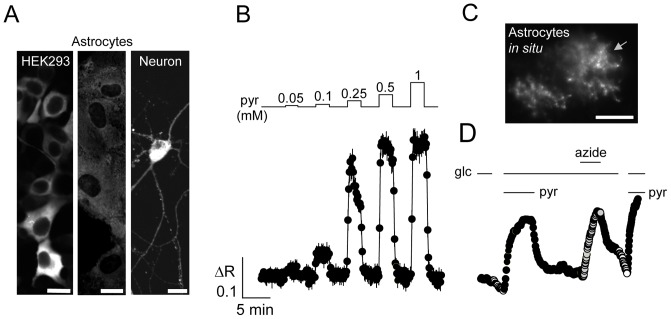
Imaging pyruvate in mammalian cells. A. Distribution of Pyronic in HEK293 cells, and astrocytes and neurons in culture. Scale bars represent 20 µm. B. Response of the fluorescence ratio to pulses of 0.05, 0.1, 0.25 0.5 and 1 mM pyruvate in HEK293 cells. C. Distribution of Pyronic in protoplasmic astrocytes in a hippocampal slice. Bar represents 20 µm. D. Response of the astrocyte arrowed in C to glucose depletions, pulses of 10 mM pyruvate and exposure to 5 mM sodium azide.

Expressed in HEK293 cells, neurons and astrocytes, Pyronic showed the expected cytosolic distribution (Fig. 3A). There was a rapid response to changes in extracellular pyruvate, reaching saturation towards the millimolar range (Fig. 3B). The maximum change in fluorescence ratio of the sensor expressed in cells was about 40%, twice as much as that observed *in vitro* (see Fig. 2), a phenomenon previously noted with a structurally related lactate nanosensor [6]. The fluorescence ratio did not respond to withdrawal of extracellular glucose (Figure S2A), indicating that in the absence of extracellular pyruvate, cytosolic pyruvate lies below the detection range of the sensor (i.e. <10 µM, see Fig. 2). This low cytosolic pyruvate is in register with the low intracellular lactate measured in cultured cells under the same conditions [6] and can be explained by loss of pyruvate and lactate through highly permeable monocarbocylate transporters (MCTs). Expression in mature hippocampal astrocytes by stereotaxic injection of an astrocyte-specific adenoviral vector, followed by preparation of acute tissue slices, revealed the typical fractal aspect of protoplasmic astrocytes as found *in situ* (Fig. 3C). In contrast to astrocytes in culture, *in situ* astrocytes showed a decrease in cytosolic pyruvate upon removal of extracellular glucose (Fig. 3D). This finding may be explained by the presence of MCT4 in mature astrocytes [7], an isoform that has a much lower affinity for pyruvate than MCT1, the isoform found in astrocytes in culture [8]–[10]. Consistently, astrocytes *in situ* were much slower than cultured astrocytes at taking up and releasing pyruvate (Fig. 3D). A caveat is that adenoviral vectors may cause local inflammation when used *in vivo*. For a systematic study, the sensor may instead be expressed by means of adeno-associated viral vectors or transgenesis. If confirmed, the existence in adult astrocytes of a permeability to pyruvate would support the hypothesis that astrocytes shuttle energy to neurons by exporting lactate in exchange for pyruvate [11], [12]. As expected from acute stimulation of glycolysis [13] and lactate production [6], adult astrocytes quickly fill up with pyruvate after mitochondrial inhibition with complex IV blocker sodium azide (Fig. 3D).

Taking advantage of the possibility of measuring the extreme points of the saturation curve in each cell by successive incubations with and without pyruvate, the sensor was calibrated by using the value of the K_D_ estimated *in vitro*, according to the equation pyruvate (mM)  =  (0.107*ΔR)/(ΔRmax – ΔR). Figure S2B shows an example of the transformation of fluorescence ratio data into pyruvate concentration using this two-point calibration protocol. Confirming previous results with a lactate FRET nanosensor [6], pyruvate uptake by HEK293 cells was fully inhibited by the specific MCT inhibitor AR-C155858 and partially by the non-specific MCT inhibitor phloretin (Fig. S3). Therefore, AR-C155858 was selected as the preferred MCT blocker for the inhibitor-stop protocols described below.

### Glycolytic pyruvate production

**Figure 4:**
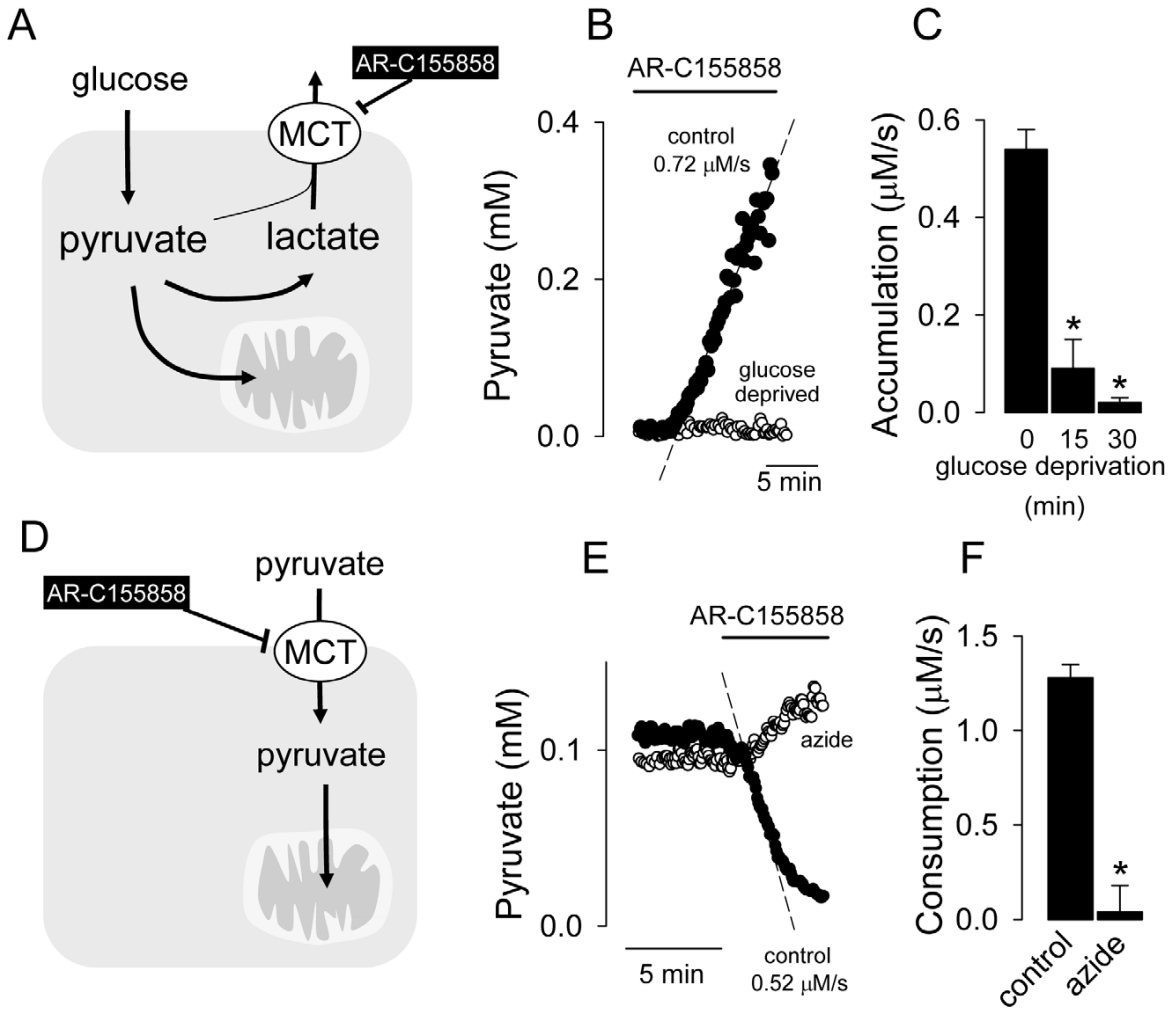
Estimation of glycolytic and mitochondrial fluxes. A. The cytosolic pyruvate pool is determined by glycolytic production, mitochondrial consumption and conversion into lactate, which then leaves the cell through the MCTs. A minor amount of pyruvate may also be lost through the MCTs. B. Accumulation of pyruvate in a HEK293 cell exposed to the MCT blocker AR-C155858 (1 µM) in the presence of 2 mM glucose (control) or in a cell deprived of glucose for 30 min. C. Effect of increasing times of glucose deprivation on the rate of pyruvate accumulation induced by 1 µM AR-C155858 in HEK293 cells. D. With pyruvate as exclusive fuel, the cytosolic pyruvate pool is only determined by influx through the MCTs and by mitochondrial consumption. E. HEK293 cells were depleted of glucose for 1 hour, and then exposed to 0.3 mM pyruvate in the absence (control) or presence of 5 mM sodium azide. Addition of 1 µM AR-C155858 resulted in a decrease in intracellular pyruvate concentration only in the control cell. F. Summary of the effect of 5 mM sodium azide on the rate of mitochondrial pyruvate consumption measured as described in E.

The intracellular concentration of pyruvate is determined by a dynamic balance between pyruvate production by glycolysis and pyruvate consumption by mitochondria, LDH and MCTs (Fig. 4A). Inhibition of pyruvate and lactate efflux by application of the MCT blocker AR-C155858 in the presence of glucose led to progressive accumulation of intracellular pyruvate, which was inhibited by glucose deprivation (Figs. 4B–C), confirming the glycolytic origin of the pyruvate. This inhibitor-stop protocol thus provides a strategy for the assessment of glycolysis at its exit point. Consistent with the time dependence of MCT-blockage described previously [14], a delay of about 2 min was observed between the application of AR-C155858 and the onset of pyruvate accumulation.

### Measurement of mitochondrial pyruvate consumption

In order to assess mitochondrial flux, HEK293 cells expressing Pyronic were first depleted of glucose for 1 hour, a condition in which glycolytic pyruvate production is negligible (Fig. 4C). Exposure of glucose-deprived cells to pyruvate resulted in a simpler dynamic equilibrium between influx from the extracellular space through the MCTs and clearance due to mitochondrial pyruvate consumption (Fig. 4D). We observed that intracellular pyruvate stabilized at a value of about 30–50% that of extracellular pyruvate. For instance in the cells illustrated in Fig. 4E, bathed in 0.3 mM pyruvate, intracellular pyruvate fluctuated around 0.1 mM. Interruption of the steady-state by MCT-blockage led to a steady decrease in cytosolic pyruvate until the pool was fully depleted. The rate of pyruvate clearance was constant over most of the concentration range, making it evident that the pyruvate transporters were saturated (Fig. 4E). Inhibition of mitochondrial oxidative phosphorylation with sodium azide blocked the clearance of pyruvate (Fig. 4E–F), confirming that this clearance is due to mitochondrial metabolism.

### Modulation of mitochondrial flux in intact brain cells

**Figure 5:**
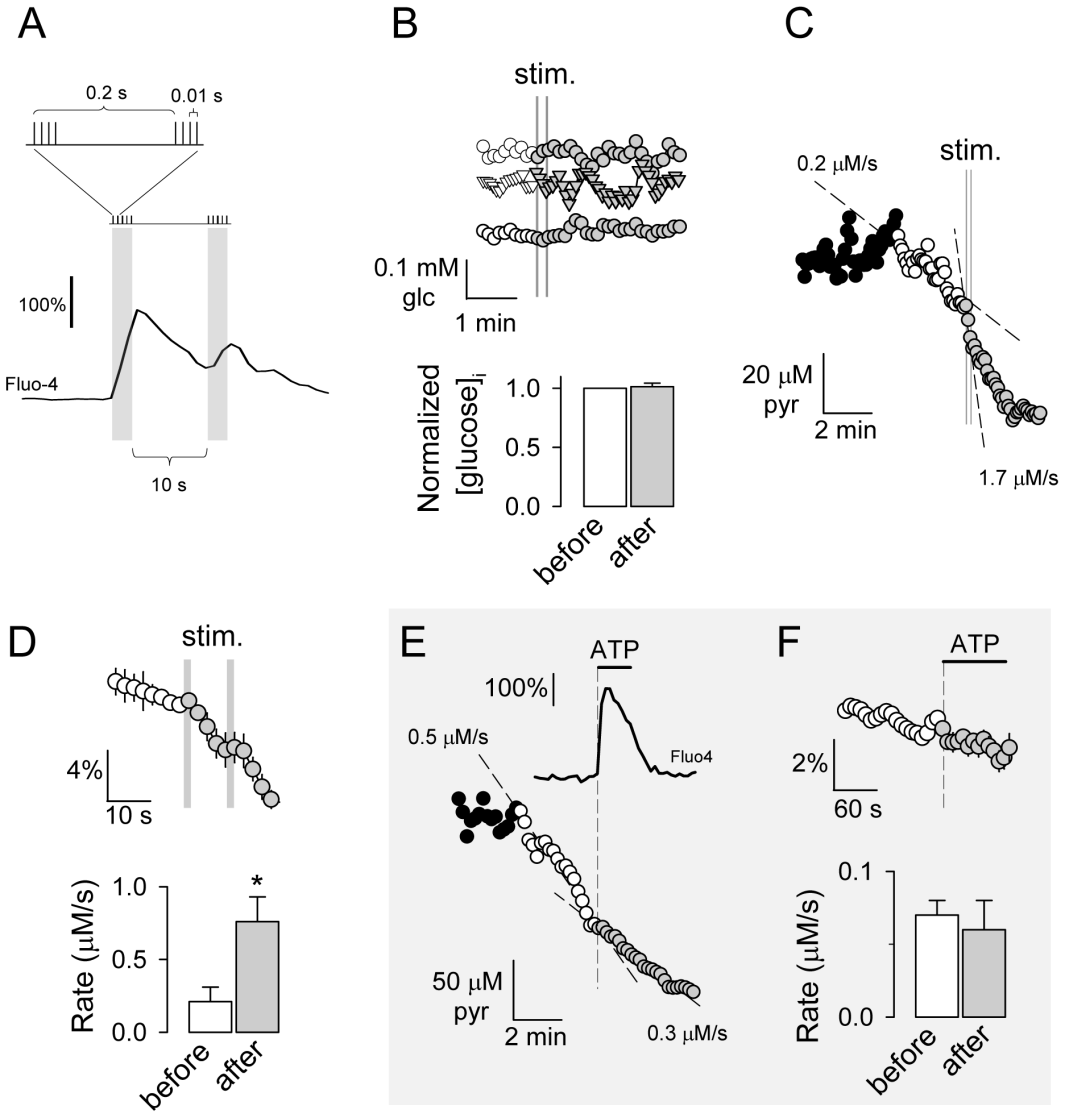
Early metabolic events during brain cell activation. A. Fluo-4-loaded hippocampal neurons were stimulated with a theta burst as described in Materials and Methods. Data are means of 8 cells. B. Effect of a theta burst on intracellular glucose in 3 neurons. Bars illustrate data from 8 cells in three experiments. C. Effect of a theta burst on the rate of mitochondrial pyruvate consumption in a neuron, measured in the presence of 0.4 mM pyruvate and 1 µM AR-C155858. D. Pooled data from 9 neurons in five experiments like that shown in C. Bars summarize the rates in the 30 s periods before and after the theta burst. E. Effect of 5 µM ATP on the mitochondrial pyruvate consumption in an astrocyte, measured in the presence of 0.4 mM pyruvate and 1 µM AR-C155858. The calcium response to 5 µM ATP in a separate experiment is also shown. Data are means from 8 cells. F. Pooled pyruvate data from 40 astrocytes in an experiment like that shown in E. Bars summarize the rates 2 minutes before and after application of ATP in 40 cells in five experiments.

The high spatial and temporal resolution afforded by fluorescence microscopy was exploited in order to look for fast metabolic events in mixed cultures of neurons and astrocytes, conditions in which both cell types differentiate best, both morphologically and metabolically [15]. First of all, cultures were field-stimulated with a short theta burst, a protocol that mimics moderate levels of hippocampal activity [16]. The effectiveness of a theta burst to activate neurons was confirmed by a strong calcium transient (Fig. 5A). Measurement with a glucose nanosensor [17] showed no changes in the neuronal glucose pool during or after the theta burst (Fig. 5B), a pool that is known to be rapidly mobilized in response to a pH change [18]. In contrast, the pyruvate sensor revealed that within seconds of the first train of impulses, the rate of mitochondrial metabolism in neurons went up by over 300% (Figs. 5C and 5D). In astrocytes, the basal rate of mitochondrial pyruvate consumption was lower than in neurons, and was not stimulated by a large calcium transient elicited with extracellular ATP (Figs. 5E and 5F). Control experiments showed that 1 µM AR-C155858 inhibited neuronal and astrocytic pyruvate transport by over 95% (data not shown). As with HEK293 cells, pyruvate clearance in both astrocytes and neurons was linear at low pyruvate levels (Figs. 5C and 5E), demonstrating high-affinity pyruvate uptake by mitochondria. The dissociation between pyruvate and glucose metabolism during the first moments of activation supports the notion that active neurons are preferentially energized by lactate and not by glucose [19]. The lack of response of astrocytic mitochondria shows that a calcium signal is not sufficient to stimulate mitochondrial metabolism, and suggests that astrocytes may not contribute significantly to the oxygen dip that follows brain activation.

## Discussion

Mitochondria are exciting. Fission-fusion dynamics, calcium dynamics, apoptosis, autophagy, interaction with the endoplasmic reticulum, roles in cancer and degenerative diseases, and so on. Many of these processes can be followed in real-time by fluorescence microscopy, but there is no practical counterpart for the main biological function of mitochondria, which is metabolism. Here we introduce a technique that quantifies mitochondrial metabolism in intact cells using fluorescence microscopy.

The pyruvate sensor responded to pyruvate between 10 µM and 1 mM, spanning the physiological range measured in plasma and mammalian tissues, and it was shown to be insensitive to pH, redox ratio, and to several closely related cytosolic and mitochondrial metabolites. Pyronic can be added to the emerging list of fluorescent metabolite sensors that have been validated in mammalian cells, which include probes for glucose [17], ATP [20], [21], NADH/NAD^+^ [12], [22], and lactate [6]. One of the positive characteristic associated to Pyronic is that it makes non-invasive calibration possible, which permits an estimation of concentration in each experiment without recourse to the harsh procedures of cell permeabilization. An important characteristic of the pyruvate sensor is that it can be combined with transport blockers to obtain quantitative estimates of metabolic flux.

The gold standard for mitochondrial function is respirometry, a technique long used for cells in suspension and isolated mitochondria. A successful refinement of respirometry was introduced recently for adherent cells in multi-well format [23]. The pyruvate sensor also measures mitochondrial rate but differs from respirometry in scope, strengths and limitations. Respirometry measures mitochondrial speed regardless of the actual substrate that is being metabolized, e.g. pyruvate, fatty acids, ketones, etc, whereas the current technique is specific for pyruvate. This restriction may be of use for the study of the mitochondrial pyruvate carrier MPC [24], [25], which was recently identified as a target for thiazolidinediones, a major class of hypoglycemic drugs [26]. In cells expressing the anaplerotic enzyme pyruvate carboxylase, the nanosensor will report the sum of flux via pyruvate dehydrogenase and via pyruvate carboxylase, routes that may be further dissected with enzyme inhibitors and/or knockdown approaches. Normal and diseased tissues are heterogeneous in terms of expression and distribution of metabolic enzymes [27], which implies cell type-specific metabolic concentrations and fluxes. A distinctive advantage of the pyruvate nanosensor is that it provides single-cell resolution, granting access to specific cell types within complex cultures and tissues. Even in monoclonal cell populations cultured under identical conditions, there is regulated cell-to-cell variability, which may be exploited to uncover novel molecular mechanisms [28]. Another advantage is high temporal resolution, giving access to rapid phenomena. These two features were combined in our study with the detection of fast metabolic events in mature neurons in the presence of astrocytes. A general limitation of the present technique is that the sensor needs to be introduced into cells, making it impractical for the study of freshly isolated cells or tissues in a clinical context, as readily occurs with respirometry. A specific limitation of the mitochondrial flux measurement is that it requires the absence of glucose, a factor that may possibly affect mitochondrial function. Altogether, the pyruvate sensor method and respirometry appear complementary, as they explore separate aspects of mitochondrial function and have different constraints.

The potential of the sensor to address specific questions was illustrated using HEK293 cells, astrocytes and neurons. A fundamental problem in bioenergetics concerns the control of mitochondrial metabolism. Experiments in isolated mitochondria have identified several candidates including cytosolic Ca^2+^, mitochondrial Ca^2+^, the ATP/ADP ratio, pyruvate availability, and the activity of the mitochondrial pyruvate carrier [1], [24], [25], [29], [30]. The constancy of the pyruvate clearance rate we observed shows that mitochondrial pyruvate transporters are saturated at physiological pyruvate levels, and that pyruvate availability is not rate limiting and unlikely to control mitochondrial metabolism in intact cells. This is an important observation, for there is no consensus in the literature about the affinity of mitochondria for pyruvate. Measurements in isolated organelles have estimated K_m_ values for mitochondrial pyruvate uptake as distant as 5 µm and 1 mM [31]–[33]. In the brain, there are other questions to be addressed, such as how fast are the responses of mitochondria to energy demand and what are the relative roles of neurons and astrocytes. In astrocytes, we found that the sizable calcium transient elicited with ATP failed to stimulate mitochondrial flux, while a similar calcium transient triggered in neurons by moderate electrical stimulation induced a strong stimulation of pyruvate consumption within seconds. Glucose levels were unaffected by electrical stimulation. Specific measurement of glucose consumption in single neurons with the FRET glucose sensor [13] may help to separate possible contributions from glucose transport and metabolism. Our results are significant for several reasons: they imply that neurons are responsible for the acute dip in oxygen concentration that follows neural activation *in situ*; they provide a metabolic explanation for the overshoot in mitochondrial NAD(P)H fluorescence that follows neuronal activation [34]; and they show that active neurons undergo a substantial increase in mitochondrial pyruvate flux without apparent changes in glucose metabolism, leaving lactate as the main source of energy for active neurons [19], [35]. The lack of response in astrocytes supports a role for mitochondria-specific factors that condition the response to calcium, such as the mitochondrial transporters aralar and citrin [30]. Further understanding of short-term mitochondrial flux regulation by calcium signals in neurons and astrocytes may require a more detailed characterization, including dose-response curves, the use of pharmacological blockers and receptor agonists, and calcium quantification with ratiometric dyes. The significant pyruvate permeability detected in astrocytes in hippocampal slices came as a surprise because the MCT isoform present in astrocytes (MCT4) displayed an exceedingly low affinity for pyruvate when expressed in oocytes [8], [9]. Whereas the nature of the pyruvate pathway in mature astrocytes remains to be investigated, its presence provides a missing link for the proposed astrocyte-neuronal redox conveyor [11], [12].

Our pyruvate sensor may also be of interest for biotechnology, a field of study requiring high resolution techniques [36]. Pyruvate is a metabolic hub, situated at the crossroads of glycolysis and mitochondrial metabolism, and is the starting metabolite for multiple cellular biosynthetic pathways. Pyruvate is a molecule of industrial interest as it is currently manufactured as a dietary complement, a weight control supplement and antioxidant, as well as being as a raw material, widely applied in the chemical, pharmaceutical, and agrochemical industries. Standard methods to measure pyruvate are based on enzymatic reactions that are monitored by means of photometric, amperometric, HPLC and other methods. Relative advantages of the sensor in this context are that it does not consume pyruvate and does not require destruction of the sample. It is also sensitive, being to date the only method capable of detecting the minute amounts of pyruvate present in a single cell. Being genetically-encoded, the pyruvate sensor can be produced in great quantities by bacteria, at low cost.

In summary, we have introduced here a genetically-encoded FRET sensor for pyruvate, as well as methods for quantitative estimation of pyruvate concentration, pyruvate transport, pyruvate production and mitochondrial pyruvate consumption in intact single cells. Demonstrating their potential, these methods revealed that astrocytes possess a significant surface permeability to pyruvate, that mitochondria are not limited by substrate availability and that a calcium transient is rapidly followed by mitochondrial flux activation in neurons, but not in astrocytes. We expect these techniques to be of great practical interest.

## Materials and Methods

Standard reagents and inhibitors were acquired from Sigma. AR-C155858 was purchased from Haoyuan Chemexpress (Shanghai).

### Construction of the pyruvate sensor

Bacterial genomic DNA was isolated using the EZNA isolation kit (Omega bio-tek). The PdhR gene from *E. coli* was amplified with specific primers. To simplify the cloning strategy, a HindIII site was removed by introducing a single-nucleotide silent mutation. For easy subcloning of PhdR sequences, restriction sites AflII and KpnI were respectively introduced at the 3′ and 5′ end of the amplicon. PCR reactions were carried out with a KOD hot start DNA polymerase (Novagen) and their products were checked by sequencing (Macrogen. Inc). Four variants of the pyruvate sensor were generated using the Gateway® Recombination Cloning Technology (Invitrogen), according to the manufactureŕs instructions. Briefly, two plasmid vectors were constructed: a destination vector and an entry vector. The destination vector (pDEST01) comprised the backbone vector pRSET-B in which the DNA sequences coding for a polyhistidine tail, mTFP and Venus were cloned downstream of the bacterial T7 promoter. A recombination cassette, amplified from pDEST14 by PCR, was intercalated between the sequences for mTFP and Venus. The entry vector was constructed using the pCR®8/GW/TOPO TA vector by cloning the sequences corresponding to the full PdhR from *E. coli* (pENTPdhR01). Finally, four expression vectors were generated by LR recombination reaction between the destination vector pDEST01 and the entry vector pENTPdhR01, followed by conditional removal of the linker between mTFP and PdhR or the linker between PdhR and Venus by digestion of the expression vectors with the restriction enzymes KpnI and AflII.

### Protein purification

The four variants of the pyruvate sensor were transformed into competent *E. coli* BL21 (DE3). A single colony was inoculated into 100 ml of LB medium with 100 mg/ml ampicillin (no IPTG) and shaken in the dark for 2–3 days. Cells were collected by centrifugation at 5000 rpm (4°C) for 10 min and disrupted by sonication (Hielscher Ultrasound Technology) in 5 mL of Tris-HCl buffer pH 8.0. A cell-free extract was obtained by centrifugation at 10,000 rpm (4°C) for 1 hour and subsequent filtering of the supernatant (0.45 µm). Proteins were purified using a nickel resin (His Bin® from Novagen) as recommended by the manufacturer. Eluted proteins were incubated overnight at 4°C, quantified using the Biuret method and stored at −20°C in 20% glycerol. The variant that showed the largest change in fluorescence ratio, which we named Pyronic (Pyruvate Optical Nano-Indicator from CECs), was cloned into pcDNA3.1(-) for expression in eukaryotic cells using the restriction sites BamHI and HindIII.

### Animals and cell cultures

Mixed F1 (C57BL/6J x CBA/J, cultures) or sv/129 male mice (stereotaxis) were kept in an animal room under Specific Pathogen Free (SPF) conditions at a room temperature of 20±2°C, in a 12/12 h light/dark cycle with free access to food and water. Procedures were approved by the Centro de Estudios Científicos Animal Care and Use Committee. Mixed cortical cultures (1–3 day-old neonates) and hippocampal cultures (17.5 day embryos) were prepared as detailed previously [13]. Astrocytes in cortical cultures were used at days 7–10 and neurons in hippocampal cultures were used at days 14–17. HEK293 cells were acquired from the American Type Culture Collection and cultured at 37°C in 95% air/5% CO_2_ in DMEM/F12 10% fetal bovine serum. Cultures were transfected at 60% confluence using Lipofectamine 2000 (Gibco) or alternatively, exposed to 5×10^6^ PFU of Ad Pyronic or Ad FLII^12^Pglu600 µΔ6 (serotype 5, custom made by Vector Biolabs), and studied after 24–72 h.

### Stereotaxis and preparation of acute hippocampal slices

Two-month old Sv/129 male mice animals were anesthetized under sterile conditions with an intraperitoneal injection of 2% avertin, and secured in a stereotactic frame (Just for Mice™, Stoelting 51725). Body temperature was maintained at 37°C with an ATC temperature controller (World Precision Instruments). After shaving, the skin was anesthetized with 4% lidocaine and cut with scissors. A 0.3 mm-diameter circular hole was made in the skull with a micro drill (Fine Science Tools). The tip of a a 34-gauge needle linked to a Hamilton syringe by a polyethylene catheter was positioned in the hippocampus, 2 mm posterior to Bregma, 1.5 mm mediolateral, and 1.8 mm below the pial surface. Ad Pyronic (1.85×10^6^ pfu diluted in 2 µl of PBS buffer supplemented with 1% BSA) was injected with a Fusion 100 Syringe Pump (Chemyx Inc.). After removing the needle, the skin was anesthetized with 4% lidocaine and sutured. After 3–4 weeks, hippocampal slices were prepared as follows. Animals were sacrificed by cervical dislocation, the head was removed and the brain was carefully extracted. Coronal brain sections at a thickness of 200 µm were prepared using a vibratome (1000 Plus Sectioning System, Vibratome®). During slicing, the brain was kept in a cold buffer (mM): 125 NaCl, 2.5 KCl, 1.25 NaH_2_PO_4_, 26 NaHCO_3_, MgCl_2_, 0.5 CaCl_2_ and 10 glucose) gassed with 95% O_2_/5% CO_2_, pH 7.4. Hippocampal slices were transferred to a second buffer (mM): 125 NaCl, 2.5 KCl, 1.25 NaH_2_PO_4_, 26 NaHCO_3_, 1 MgCl_2_, 1.5 CaCl_2_, 0.5 lactate and 10 glucose) gassed with 95% O_2_/5% CO_2_, pH 7.4, at room temperature. During experiments, slices were superfused with a 95% O_2_/5% CO_2_-gassed KRH buffer (mM): 126 NaCl, 3 KCl, 1.25 NaH_2_PO_4_, 1.25 CaCl_2_, 1.25 MgCl_2_, 10 glucose and 26 NaHCO_3_, pH 7.4.

### Fluorescence measurements

Nickel-purified proteins were resuspended at 100 nM in an intracellular buffer containing (mM): 10 NaCl, 130 KCl, 1.25 MgSO_4_ and 10 HEPES, pH 7.0, and measured with a microplate reader analyzer (EnVision, PerkinElmer). The proteins were excited at 430 nm and the intensity of fluorescence emission of mTFP and Venus were recorded at 485 nm and 528 nm, respectively. The ratio between the emissions was used to characterize the sensors. Emission spectra were obtained at 430 nm excitation, with 2 nm windows. Cells were imaged at room temperature (22−25°C) in a 95% air/5% CO_2_-gassed KRH-bicarbonate buffer of the following composition (in mM): 112 NaCl, 1.25 CaCl_2_, 1.25 MgSO_4_, 1-2 glucose, 10 HEPES, 24 NaHCO_3_, pH 7.4, with 3 mM KCl (brain cells) or 5 mM KCl (HEK293) using an upright Olympus FV1000 confocal microscope equipped with a 20X water immersion objective (NA 1.0) and a 440 nm solid-state laser. Alternatively, cells were imaged with an Olympus IX70 or with an Olympus BX51 microscope equipped with a 40X oil-immersion objective (NA 1.3) or with a 20X water-immersion objective (NA 0.95). Microscopes were equipped with Cairn monochromators (Faversham, UK), and either a Hamamatsu Orca camera controlled by Kinetics software or a Rollera camera controlled with Metafluor software, respectively. For nanosensor ratio measurements, cells were excited at 430 nm for 0.2–0.8 s. Emission was divided with a Cairn Optosplit, equipped with band pass filters at 480±20 (mTFP) and 535±15 nm (Venus). The ratio between mTFP and Venus was used to estimate pyruvate concentration according to the calibration procedure described in the Results section (Fig. S2). Intracellular glucose concentration was measured with the FRET nanosensor FLII^12^glu700 µΔ6, which has a K_D_ for glucose of 660±160 [17].

### Electrical stimulation

Mixed cultures of hippocampal neurons and astrocytes grown on 25 mm coverslips were field-stimulated using a RC-21BRFS chamber (Warner Instruments) and a WPI PRO-4 device. Pulses (50 mA output) were generated with a WPI A385 High Current Stimulus Isolator connected to a WPI A382 Battery Charger. The stimulation protocol, illustrated in Fig. 5A, was a short theta burst, a protocol that mimics moderate hippocampal activity [16]. It consists of two trains of impulses separated by 10 seconds. Each train lasts for 1 second and is composed of twenty 1 ms pulses, distributed into five groups of four pulses.

### Calcium measurement

Cytosolic calcium was estimated with Fluo-4 (Invitrogen). The dye was loaded in the AM form (4 µM) for 30 minutes at 37°C in KRH buffer without bicarbonate and supplemented with 2 mM glucose and 1 mM lactate. After loading, cultures were transferred to the standard KRH-bicarbonate buffer and left to rest for another 15 min prior to measurements.

### Statistical analysis

Unless otherwise specified, experiments were repeated three to five times, with 6–12 cells per experiment. Error bars represent SEM. Regression and statistical analyses were carried out with the computer program SigmaPlot (Jandel). Differences in mean values of paired samples were evaluated with the Students t-test. Differences between more than two groups were evaluated with the Kruskal-Wallis one way analysis of variance on ranks followed by Dunns test. P values <0.05 were considered significant and are indicated with an asterisk (*).

## Supporting Information

Figure S1 **Related to** **Fig. 1** **.** DNA and amino acid sequences of the pyruvate sensors. A. DNA sequences of four variants of the pyruvate sensor. B. Amino acid sequences of four variants of the pyruvate sensor. Variant 3 was termed Pyronic.
(DOC)

Figure S2 **Related to** **Fig. 2** **.** Two-point calibration of Pyronic in HEK293 cells. A. Removal of glucose and lactate had no detectable effect on the fluorescence ratio of Pyronic, indicating that resting pyruvate levels are below the sensitivity of the sensor (i.e. <10 µM). The response to 1 mM pyruvate is shown. Data are means ± SEM from 8 cells. B. A cell was sequentially exposed to 1 mM and 0.4 mM pyruvate. The lower panel shows the fluorescence ratio, where R_0_ is the ratio in the absence of pyruvate and ΔR_max_ is the difference between R_0_ and the maximum ratio estimated in 1 mM pyruvate. The value of the ratio at each time point minus R_0_, ΔR, was transformed into pyruvate concentration (mM, top panel) using the measured ΔR_max_ and the K_D_ estimated *in vitro* (107 µM, see Fig. 2B).
(DOC)

Figure S3 **Related to** **Fig. 4** **.** Inhibition of pyruvate transport in HEK293 cells. A. Time course of Pyronic fluorescence ratio in a single cell sequentially exposed to 1 mM pyruvate alone, to 1 mM pyruvate in the presence of 50 µM phloretin (5 min preincubation) and to 1 mM pyruvate in the presence of 1 µM AR-C155858 (5 min preincubation). B. The same data as in A, expressed as pyruvate concentration. C. Summary of 24 cells in three separate experiments. Mean ± SEM.
(DOC)
