# Supplementary material for: Imaging Mitochondrial Flux in Single Cells with a FRET Sensor for Pyruvate
Source: PLoS One. 2014 Jan 21;9(1):e85780. doi: 10.1371/journal.pone.0085780 (PMC3897509; doi:10.1371/journal.pone.0085780)
Supplement: Figure S1 — Related to Fig. 1 . DNA and amino acid sequences of the pyruvate sensors. A. DNA sequences of four variants of the pyruvate sensor. B. Amino acid sequences of four variants of the pyruvate sensor. Variant 3 was termed Pyronic. (DOC) [file pone.0085780.s001.doc]

**Figure S1, related to Fig. 1.** **Part A. DNA sequences of 4 variants of the pyruvate sensor.**


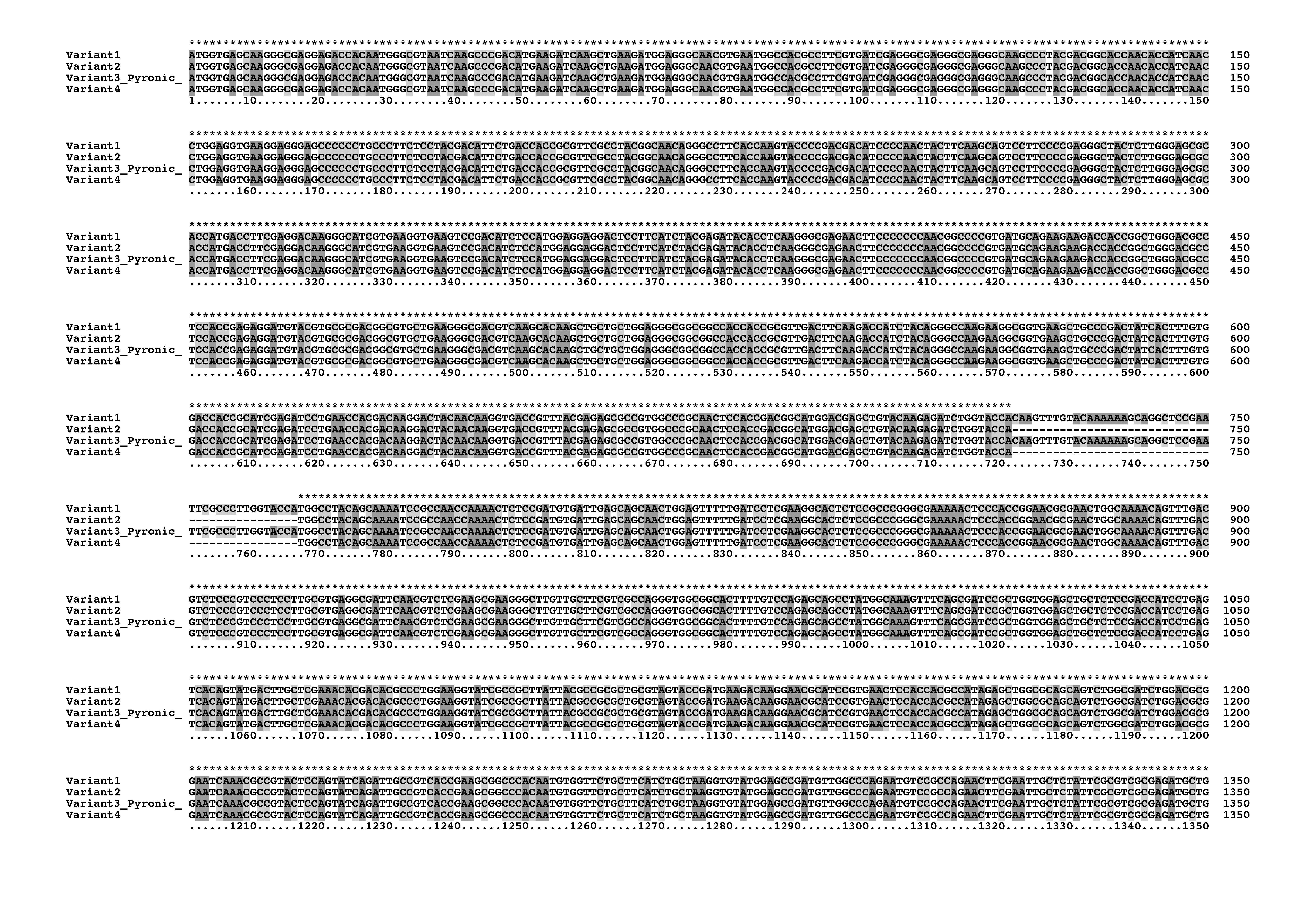


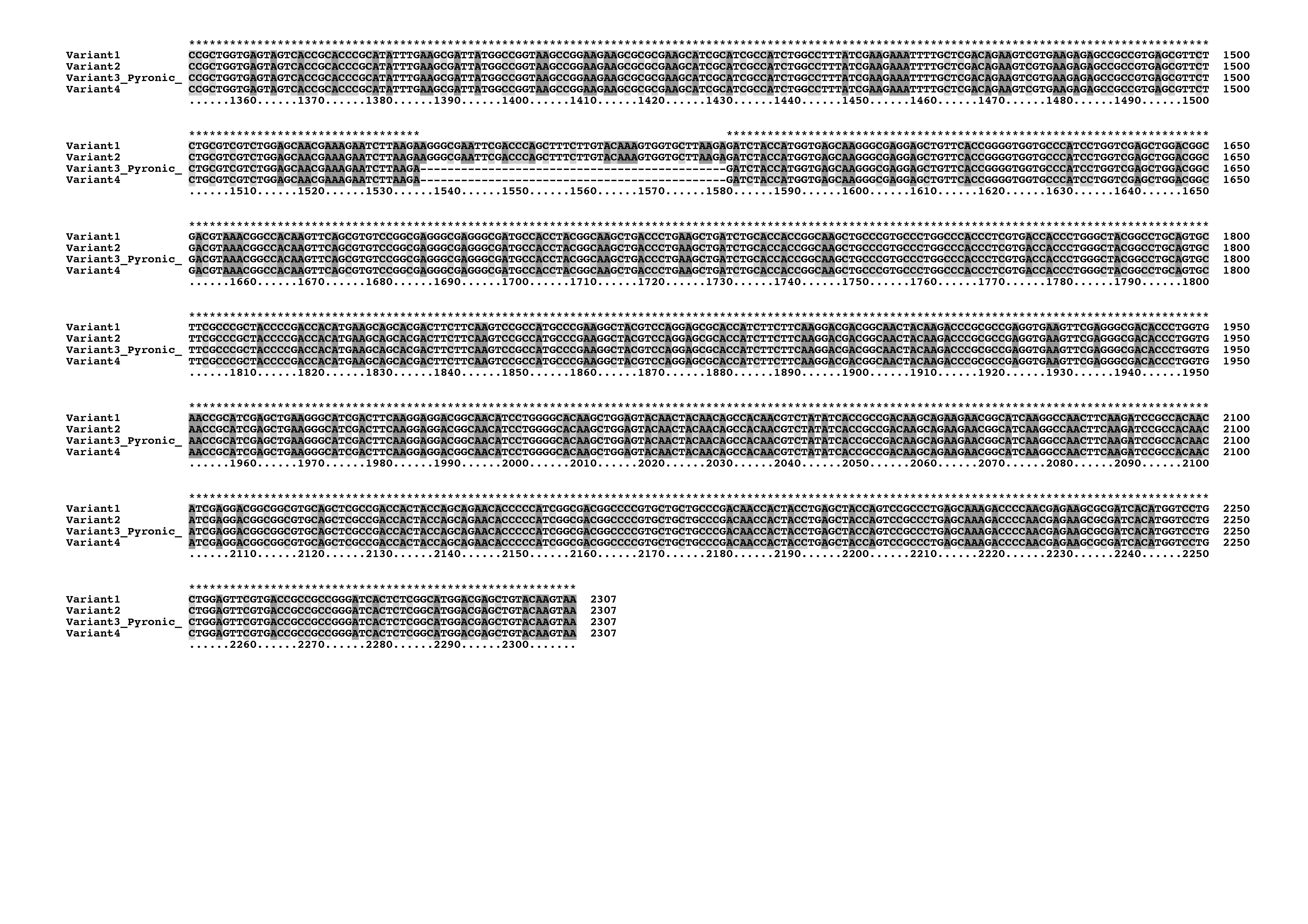


**Figure S1, related to Fig. 1.** **Part B. Amino acid sequences of 4 variants of the pyruvate sensor.**


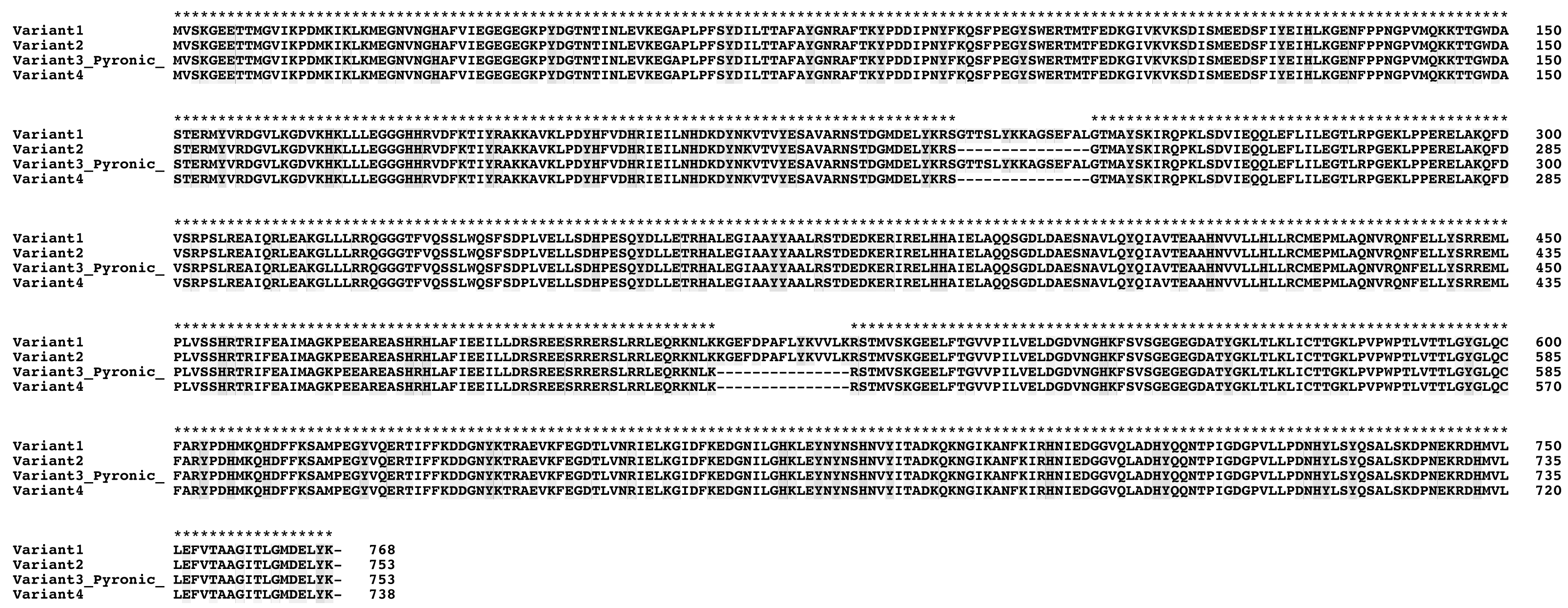


**Figure S1, related to Fig. 1. DNA and amino acid sequences of the pyruvate sensors.** A. DNA sequences of four variants of the pyruvate sensor. B. Amino acid sequences of four variants of the pyruvate sensor. Variant 3 was termed Pyronic.
