# Supplementary material for: Imaging Mitochondrial Flux in Single Cells with a FRET Sensor for Pyruvate
Source: PLoS One. 2014 Jan 21;9(1):e85780. doi: 10.1371/journal.pone.0085780 (PMC3897509; doi:10.1371/journal.pone.0085780)
Supplement: Figure S2 — Related to Fig. 2 . Two-point calibration of Pyronic in HEK293 cells. A. Removal of glucose and lactate had no detectable effect on the fluorescence ratio of Pyronic, indicating that resting pyruvate levels are below the sensitivity of the sensor (i.e. <10 µM). The response to 1 mM pyruvate is shown. Data are means ± SEM from 8 cells. B. A cell was sequentially exposed to 1 mM and 0.4 mM pyruvate. The lower panel shows the fluorescence ratio, where R0 is the ratio in the absence of pyruvate and ΔRmax is the difference between R0 and the maximum ratio estimated in 1 mM pyruvate. The value of the ratio at each time point minus R0, ΔR, was transformed into pyruvate concentration (mM, top panel) using the measured ΔRmax and the KD estimated in vitro (107 µM, see Fig. 2B). (DOC) [file pone.0085780.s002.doc]

**Figure S2, related to Fig. 3. Two-point calibration of Pyronic in HEK293 cells.**

**Figure S2, related to Fig. 3. Two-point calibration of Pyronic in HEK293 cells.** A. Removal of glucose and lactate had no detectable effect on the fluorescence ratio of Pyronic, indicating that resting pyruvate levels are below the sensitivity of the sensor (i.e. < 10 μM). The response to 1 mM pyruvate is shown. Data are means ± SEM from 8 cells. B. A cell was sequentially exposed to 1 mM and 0.4 mM pyruvate. The lower panel shows the fluorescence ratio, where R_0_ is the ratio in the absence of pyruvate and ΔR_max_ is the difference between R_0_ and the maximum ratio estimated in 1 mM pyruvate. The value of the ratio at each time point minus R_0_, ΔR, was transformed into pyruvate concentration (mM, top panel) using the measured ΔR_max_ and the K_D_ estimated *in vitro* (107 μM, see Fig. 2B).
