# Supplementary material for: Imaging Mitochondrial Flux in Single Cells with a FRET Sensor for Pyruvate
Source: PLoS One. 2014 Jan 21;9(1):e85780. doi: 10.1371/journal.pone.0085780 (PMC3897509; doi:10.1371/journal.pone.0085780)
Supplement: Figure S3 — Related to Fig. 4 . Inhibition of pyruvate transport in HEK293 cells. A. Time course of Pyronic fluorescence ratio in a single cell sequentially exposed to 1 mM pyruvate alone, to 1 mM pyruvate in the presence of 50 µM phloretin (5 min preincubation) and to 1 mM pyruvate in the presence of 1 µM AR-C155858 (5 min preincubation). B. The same data as in A, expressed as pyruvate concentration. C. Summary of 24 cells in three separate experiments. Mean ± SEM. (DOC) [file pone.0085780.s003.doc]

**Figure S3, related to Fig. 4. Inhibition of pyruvate transport in HEK293 cells.**

**Figure S3, related to Fig. 4. Inhibition of pyruvate transport in HEK293 cells.** A. Time course of Pyronic fluorescence ratio in a single cell sequentially exposed to 1 mM pyruvate alone, to 1 mM pyruvate in the presence of 50 μM phloretin (5 min preincubation) and to 1 mM pyruvate in the presence of 1 μM AR-C155858 (5 min preincubation). B. The same data as in A, expressed as pyruvate concentration. C. Summary of 24 cells in three separate experiments. Mean ± SEM.
